# Contact Dynamic Behaviors of Magnetic Hydrogel Soft Robots

**DOI:** 10.3390/gels11010020

**Published:** 2024-12-31

**Authors:** Yunian Shen, Yiming Zou

**Affiliations:** Department of Mechanics and Engineering Science, School of Physics, Nanjing University of Science and Technology, Nanjing 210094, China; 121113011311@njust.edu.cn

**Keywords:** magnetic hydrogel, robots, multi-field coupling, large deformation, parameter optimization

## Abstract

Magnetic hydrogel soft robots have shown great potential in various fields. However, their contact dynamic behaviors are complex, considering stick–slip motion at the contact interface, and lack accurate computational models to analyze them. This paper improves the numerical computational method for hydrogel materials with magneto-mechanical coupling effect, analyses the inchworm-like contact motion of the biomimetic bipedal magnetic hydrogel soft robot, and designs and optimizes the robot’s structure. In the constitutive model, a correction factor representing the influence of the direction of magnetic flux density on the domain density has been introduced. The magnetic part of the Helmholtz free energy has been redefined as the magnetic potential energy, which can be used to explain the phenomenon that the material will still deform when the magnetic flux density is parallel to the external magnetic field. The accuracy of the simulation is verified by comparing numerical solutions with experimental results for a magnetic hydrogel cantilever beam. Furthermore, employing the present methods, the locomotion of a magnetic hydrogel soft robot modeled after the inchworm’s gait is simulated, and the influence of the coefficient of friction on its movement is discussed. The numerical results clearly display the control effect of the external magnetic field on the robot’s motion.

## 1. Introduction

In the development of modern robotics, soft robots have become a hot topic of research due to their unique flexibility and adaptability. In recent years, soft robotics technology has undergone significant development, continuously breaking through the capabilities of traditional robotics [1]. From the pioneering work between 2009 and 2012 to the present, the field has made groundbreaking progress in actuation mechanisms, sensing technology, modeling, and control strategies [1]. Moreover, the diversity in material selection, actuation technology, sensor integration, and control strategies for soft robots provides a broad space for innovative design [2]. These technological advancements have not only propelled the development of soft robotics technology but also brought new perspectives on the types of tasks robots can perform [3]. In particular, magneto-controlled hydrogel soft robots, which combine the flexibility of soft materials with the controllability of magnetic materials, show great potential in fields such as precision manipulation [4] and biomedical engineering [5]. For instance, stomach-rolling capsule robots have been designed for biopsy and drug delivery [6], and self-folding origami robots have been developed to navigate the intestines, repair wounds, and remove ingested objects [7]. Thread-like robots have been crafted with the potential to navigate the brain’s vasculature for stroke or aneurysm treatment [8], and magnetic catheter robots have been engineered for minimally invasive cardiac procedures and lung airway inspections [9,10].

These robots can achieve precise motion control under the action of external magnetic fields, including complex path tracking and multimodal motion [11]. The prediction of the dynamic behavior of magneto-controlled hydrogel soft robots is essential in their design, manufacturing, and precise operation. However, due to the coupling effects of multiple physical fields, the nonlinearity of materials, the complexity of structures, the geometric nonlinearity [12] of large deformations, and the contact nonlinearity at interfaces, few constitutive models can accurately express their mechanical behavior, making the dynamic modeling and numerical computation methods of magneto-controlled hydrogel soft robots still a challenge [13].

Understanding and predicting the behavior of magneto-controlled hydrogel soft robots is crucial, and the related dynamic calculation methods remain a hot topic of research to this day. These methods involve not only the stress–strain relationship of materials but also the deformation and motion response under the influence of external magnetic fields [14]. In recent years, researchers have proposed various computational models, including models based on continuum mechanics and machine learning prediction models, to improve the understanding and prediction of the dynamic behavior of such robots [15]. For instance, Huang et al. (2024) proposed a hybrid modeling framework based on Absolute Nodal Coordinate Formulation (ANCF) and machine learning, which effectively predicts the dynamic response of soft robots under different working conditions [16]. Additionally, modeling methods based on Cosserat beam theory have been used to describe the kinematics and dynamics of soft robots, providing theoretical support for achieving more precise control and optimized design [17]. With the advancement of computational capabilities and the development of new materials, research on the dynamic computation of magneto-controlled hydrogel soft robots continues to deepen. Researchers have explored the impact of different magnetic field strengths and material properties on the robot’s motion through a combination of numerical simulation and experimental validation [18]. Existing models for magnetoactive soft materials are predominantly centered on magnetorheological elastomers or ferrogels, which are not directly applicable to the emerging class of hard-magnetic soft materials featuring programmed magnetic domains. In light of recent advancements in the fabrication techniques for shape-programmable magnetic soft materials [19], there is a pressing need for a systematic theoretical framework, the development of constitutive laws, and a computational approach tailored to hard-magnetic soft materials. Some scholars [20] have employed nonlinear field theory to describe the coupling of finite deformation with magnetic fields, and they argue that these materials necessitate the formulation of a new constitutive model for the ideal hard-magnetic soft material. Additionally, some scholars have conducted experiments on hydrogel soft robots under different magnetic field conditions, verifying changes in their motion patterns and dynamic characteristics [21]. These studies have deepened the understanding of the dynamic behavior of magneto-controlled hydrogel soft robots and provided an important theoretical foundation for their design and control in practical applications [22]. However, in general, the motion laws of the robots are not yet clear, and their contact mechanical behavior, in particular, is difficult to predict. Most current research still relies on experience to trial-manufacture simple-shaped magnetic hydrogel soft robots, and the design work lacks a rigorous forward design optimization process.

The object of this paper is to improve the dynamic modeling method of magnetic soft matter to analyze inchworm-like walking dynamics, complicated contact modes, and the frictional effect of the biomimetic bipedal magnetic hydrogel soft robot. The large deformation, geometric nonlinearity, and magneto-mechanical coupling of both the local contact zone and the whole compliant structure are considered. The correctness of the proposed method is validated by comparing numerical solutions with the experimental data. The remainder of this paper is organized as follows: in Section 2, the modeling and formulation for dynamics of ferromagnetic particle-embedded hydrogel soft matter are given. The magneto-mechanically coupled constitutive equations are derived. The dynamic equilibrium differential equations and their weak form are provided. In Section 3, two numerical examples are calculated to validate the present numerical computational method. In Section 4, the present method is applied to design the magnetically actuated hydrogel soft robots. The inchworm-like contact motion of the biomimetic bipedal magnetic hydrogel soft robot is analyzed. Furthermore, the influence of the coefficient of friction on dynamic behaviors is also discussed.

## 2. Modeling and Formulation for Dynamics of Ferromagnetic Particle-Embedded Hydrogel Soft Matter

### 2.1. Ferromagnetic Particle-Embedded Hydrogel

**Figure 1:**
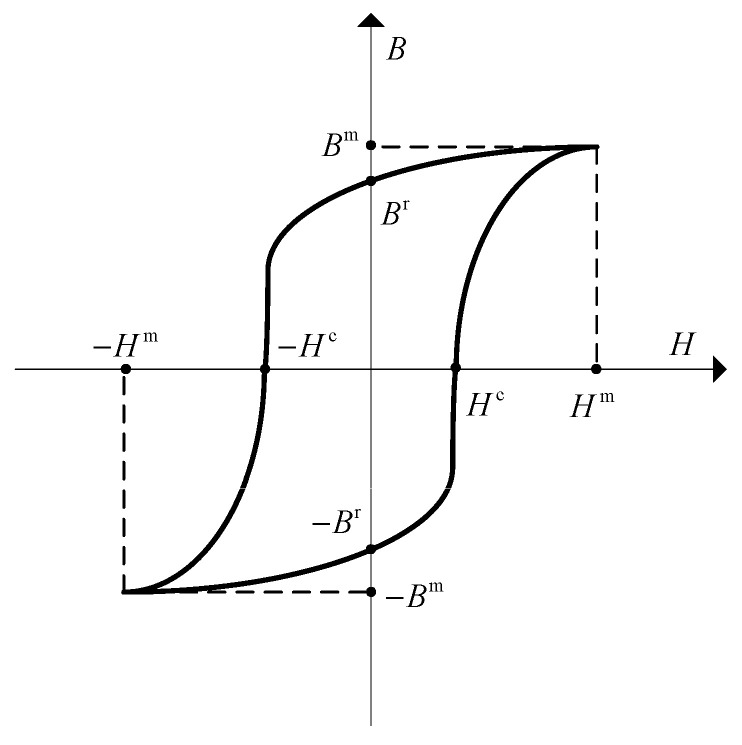
Magnetic hysteresis loops of magnetic materials along certain direction (where *H* represents the projection of the magnetic field strength **H**, *B* represents the projection of the magnetic flux density **B**, *B* represents the projection of the coercivity **H**^c^, *B*^m^ represents the projection of the saturation magnetic flux density **B**^m^, and *B*^r^ represents the projection of the remanence of **B**^r^ along a certain direction).

Hard magnetic materials and soft magnetic materials are two categories of magnetic materials with distinct magnetic properties. They exhibit significant differences in their magnetization behavior under the influence of an external magnetic field and in their application domains. Hard magnetic materials possess high coercivity $H^{c}$ and saturation magnetic flux density $B^{m}$, enabling them to maintain a strong magnetization state that is not easily altered or demagnetized by external magnetic fields. In contrast, soft magnetic materials have lower coercivity and saturation magnetic flux density. They can easily become magnetized and demagnetized under the influence of an external magnetic field, with their magnetic properties responding rapidly to changes in the external magnetic field. In addition to the differences in coercivity and saturation magnetic flux density, hard magnetic materials and soft magnetic materials also exhibit significant distinctions in other aspects [20] (see Figure 1). Hard magnetic materials have a higher remanence, meaning they can maintain a strong magnetization state even after the external magnetic field is removed, whereas soft magnetic materials have a relatively lower remanence. Furthermore, hard magnetic materials have a lower magnetic permeability while soft magnetic materials have a higher magnetic permeability. Soft magnetic materials typically have lower magnetic hysteresis loss and eddy current loss, enabling them to more effectively transform and conduct magnetic energy.

This paper selects the hard magnetic particle neodymium–iron–boron (NdFeB) magnetic powder as the magnetic additive for magnetic hydrogel materials. Firstly, neodymium–iron–boron (NdFeB) magnetic powder has a high saturation magnetic flux density, which can achieve a higher degree of magnetization under the action of an external magnetic field, thereby enhancing the deformation response ability of magnetic hydrogel materials under external magnetic field stimulation. This allows the hydrogel soft robots based on it to have a reduced requirement for external magnetic field strength to a certain extent. Secondly, these hard magnetic particles have a high coercivity, requiring a larger external magnetic field to change their magnetization state, thus maintaining the stability and reliability of the magnetic hydrogel materials. In other words, the magnetic flux density of the hard magnetic particle hydrogel soft materials with neodymium–iron–boron (NdFeB) magnetic powder as the magnetic particles will only change due to their own deformation and will not be affected by external magnetic fields changing the magnetization state of NdFeB. In addition, neodymium–iron–boron magnetic powder has good corrosion resistance and wear resistance, which can maintain magnetism for a long time in harsh environments. This allows it to maintain its own magnetism even in hydrogel materials with high water content, which enables us to assume that the magnetic hydrogel is an invariant during numerical calculations, thus decoupling the external magnetic field from its own initial configuration’s residual magnetic flux density, reducing the complexity of the constitutive model.

Hard magnetic particle hydrogels are a type of composite material that possesses the characteristics of hard magnetic materials while maintaining the soft characteristics of the superelastic body of hydrogels by adding hard magnetic particles to hydrogels. The characteristics of hard magnetic materials allow the modulus of the magnetic flux density of the material itself to be neglected in the constitutive model under the influence of an external magnetic field, making the constitutive model simpler and reducing the amount of calculation (it should be noted that the assumption of neglecting the modulus of the magnetic flux density is based on the premise that the distribution density of the magnetic particles remains constant during the deformation of the hydrogels). Its inherent softness also ensures its ability to undergo large deformation movements under external magnetic field stimulation. In summary, hard magnetic particle hydrogels are an excellent base material for magnetic hydrogel soft robots.

### 2.2. Constitutive Model

The mechanical behavior of magnetic hydrogel materials is influenced by magnetic fields; therefore, studying the magneto-thermo-mechanically coupled constitutive equations is crucial for understanding and predicting the material’s deformation and deformation processes. The constitutive equations describe the stress–strain relationship of the material and provide a numerical calculation method for the material’s mechanical behaviors. By studying the magneto-thermo-mechanically coupled constitutive equations of magnetic hydrogel materials and incorporating them into simulation models, accurate modeling and prediction of the mechanical behavior of soft robots under various working conditions can be achieved. This helps optimize the design and control strategies of the robots, enhancing their performance and adaptability.

(1) Stretching of material line element

**Figure 2:**
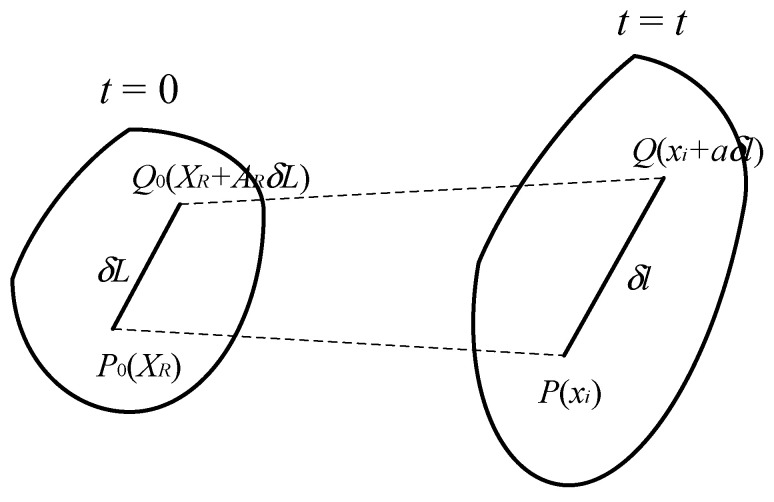
Schematic diagram of material line elements.

Hydrogel materials are a class of deformable bodies that can undergo significant changes in shape. When an object deforms, the distance between material particles changes. In continuum mechanics, to study the stretching and orientation changes of material line elements within a substance, as shown in Figure 2, let us consider a micro-element line segment in the reference configuration denoted as $P_{0}Q_{0}$, with its length δL and direction along the unit vector A. Let the coordinates of $P_{0}$ be X, then the coordinates of $Q_{0}$ are X+AδL. The material particles on the line segment $P_{0}Q_{0}$ undergo motion

(1) x=x(X,t)

When the material particles reach their position PQ in the current configuration, let the coordinates of P be x, the length of PQ be δl, and the direction be the direction of the unit vector a, then the coordinates of Q are x+aδl, and thus, we have

(2) x=x(X,t), x+aδl=x(X+AδL,t)

The original position of a material particle is typically represented by the coordinates X, and the new position to which the particle has moved after a period of time *t* is denoted by x. In continuum mechanics, the expression for the deformation gradient in three-dimensional space is given by the following equation:

(3) $$F_{iR}=\frac{\partial x_{i}}{\partial X_{R}}(i,R=1,2,3)$$

At this point, the length δl and the unit vector a of the material line element in the current configuration can be calculated using the deformation gradient matrix F. The calculation formulas are as follows:

(4) $$\lambda^{2}=A^{T}F^{T}FA$$

(5) $$a=\lambda^{-1}FA$$

in which λ=dl/dL represents the stretch ratio of the material line element. The concept of the stretch transformation of the material line element will be utilized in the subsequent definition of the magnetic flux density B in the current configuration of the magnetic hydrogel.

(2) Transformation of material properties between different configurations.

From a mathematical perspective, F is the Jacobian matrix that transforms from X to x, hence its determinant J=det(F) is the local volume ratio factor. Hyperelastic materials generally possess incompressibility, and hydrogels, as a typical type of hyperelastic material, allow us to consider J as a constant value of 1.

In continuum mechanics, the Cauchy stress tensor (or true stress tensor) and the first Piola–Kirchhoff stress tensor (or nominal stress tensor) are typically denoted by σ and P, respectively, and their relationship is given by the following equations:

(6) $$\sigma =J^{-1}PF^{T}\;\mathrm{and}\;P=J\sigma F^{T}$$

Since the magnetic flux density vector of magnetic materials also changes with the deformation of the object, we must also consider the relationship between the magnetic flux density of magnetic materials in the current configuration and that in the initial configuration. Similar to the change in position coordinates, we denote the magnetic field strength of the material in the current configuration as H, the magnetic flux density as Β, the magnetic field strength of the material in the reference configuration as $\tilde{H}$, and the magnetic flux density as $\tilde{Β}$. They have a relationship $B=\mu_{0}H$, where $\mu_{0}$ is the vacuum permeability. Since the permeability of hydrogel materials is essentially consistent with the vacuum permeability, the vacuum permeability can be used to represent the material’s permeability. In Zhao’s theory [20], the transformation of these two physical quantities between the reference and current configurations can be calculated using the following relationship:

(7) $$H=F^{-T}\tilde{H}\;\mathrm{and}\;\tilde{H}=F^{T}H$$

(8) $$B=J^{-1}F\tilde{B}\;\mathrm{and}\;\tilde{B}=JF^{-1}B$$

In the following text, parameters with sign ∼ are considered to be the material properties in the reference configuration, while identical expressions or symbols without the superscript represent the corresponding material properties in the current configuration.

The transformation equation can be viewed as equating the magnetic flux density of the material to a material line element for the transformation from $\tilde{Β}$ to Β. If we use the unit vectors a and A to represent the directions of Β and $\tilde{Β}$, respectively, and let $\left|Β\right|$ and $\left|\tilde{Β}\right|$ represent the magnitudes of Β and $\tilde{Β}$, then if the change in magnetic flux density is the same as that of the material line element, the changes in the unit vectors and lengths of the material line element can be calculated as follows:

(9) $${\left(\frac{\left|B\right|}{\left|\tilde{B}\right|}\right)}^{2}=A^{T}F^{T}FA$$

(10) $$a={\left(\frac{\left|B\right|}{\left|\tilde{B}\right|}\right)}^{-1}FA$$

In Equation (9), the ratio of $\left|Β\right|$ to $\left|\tilde{Β}\right|$ is considered the stretch ratio λ during the change of the material line element. If the volume ratio is neglected, then Equation (10) corresponds to the transformation formula in Zhao’s theory. This means that if we assume that in the initial configuration, there is a material line element with the same position vector as $\tilde{Β}$, and its length is equal to $\left|\tilde{Β}\right|$, then they will undergo the same changes. However, in reality, the length modulus of this material line element in the current configuration will often not be equal to $\left|\tilde{Β}\right|$.

**Figure 3:**
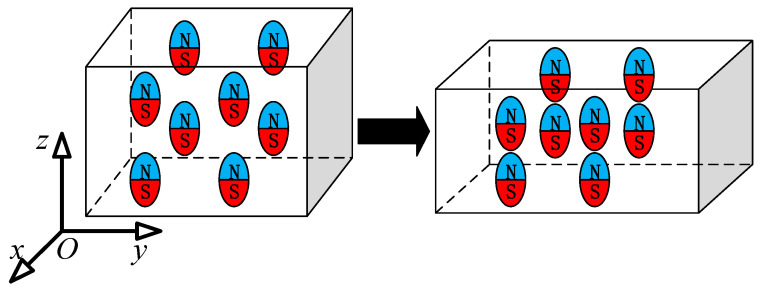
Schematic diagram of the variation of magnetic domain density with the deformation of magnetic hydrogel.

**Figure 4:**
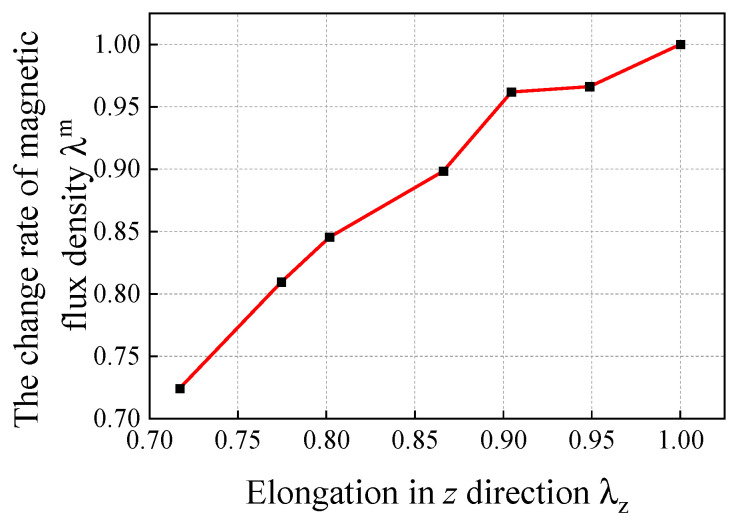
Curve of the rate of change of magnetic flux density $\lambda^{m}$ versus the material stretch ratio $\lambda_{z}$ in the same direction.

As shown in Figure 3, this paper posits that during the deformation process of magnetic hydrogel, its magnetic flux density is influenced not only by the changes in the cross-sectional area perpendicular to the direction of the magnetic flux but also by the magnetic domain density along the direction of the magnetic flux. If the influence of the magnetic domain density along the direction of the magnetic flux is neglected, then the ratio of $\left|Β\right|$ to $\left|\tilde{Β}\right|$ can be regarded as the stretch ratio of the material line element λ at the same position during the change process. To study the relationship between the rate of change of the magnetic flux density modulus and the stretch rate of the material line element, this paper defines the ratio of $\left|B\right|/\left|\tilde{B}\right|$ as $\lambda^{m}$, and measures $\lambda^{m}$ under different conditions of λ through experiments. This paper deforms the material by applying uniaxial tension along the *y*-direction, which is perpendicular to the magnetic flux density, using a sample. Since hydrogels are hyperelastic materials, it is assumed that $\lambda_{x}\lambda_{y}\lambda_{z}=1$, and $\lambda_{x}=\lambda_{y}=\lambda_{z}^{-1/2}$. In this experiment, it can be considered that the volume remains constant. In the experiment, the hydrogel samples are fixed at both ends to the claws of a carbon fiber vernier caliper, and while stretching the sample with the caliper, the dimension of the hydrogel sample in the *y*-direction can be read instantly, thereby determining $\lambda_{x}$, $\lambda_{y}$ and $\lambda_{z}$. Meanwhile, a Tesla meter is used to measure the magnetic flux density at the center of the hydrogel sample, thus obtaining the $\lambda_{z}$ of the hydrogel sample under different conditions of $\lambda^{m}$ (see Figure 4). The detailed information on the experimental sample is the same as that in Section 3.2.

When the material is stretched in the direction of the magnetic flux density, the reduction of the magnetic domain density in that direction will cause the rate of increase of the magnetic flux density $\lambda^{m}$ to be less than the elongation ratio of the material λ in that direction. Conversely, when the material is compressed, the increase in the magnetic domain density in the direction of the magnetic flux density will cause the ratio of decrease of the magnetic flux density $\lambda^{m}$ to be greater than the elongation ratio of the material λ in that direction. Therefore, this paper sets the expression as $\lambda^{m}=1+(\lambda -1)\alpha$, where α is the correction factor, which is used to quantify the deviation between the rate of change in magnetic flux density and the deformation rate due to changes in magnetic domain density. In other words, the correction factor is used to describe the change in the magnetic domain density in the direction of the magnetic flux density during deformation. Its value is determined through experimental fitting. Based on experimental data, when $\left|\left(\lambda -1\right)\right|$ is less than 0.25, α is calculated to be 0.78. The expression for the magnetic flux density B in the current configuration is corrected to the following formula:

(11) $$B=\frac{\lambda^{m}}{\lambda }J^{-1}F\tilde{B}=(\frac{1-\alpha }{\lambda }+\alpha )J^{-1}F\tilde{B}$$

in which $\lambda^{m}$ is a function of λ. For the convenience of calculation in this paper, $\lambda^{m}/\lambda$ is considered as an external constant that varies with λ and does not participate in the calculations related to the deformation gradient matrix. It can be seen that when the correction factor is 1, this transformation is consistent with Zhao’s theory.

(3) Constitutive equations

The constitutive models of materials are typically constructed based on the form of Helmholtz free energy. By making appropriate approximations or parameterizations of the Helmholtz free energy, one can obtain equations that describe the specific thermodynamic properties of materials. For instance, in solid materials, the elastic potential energy density function can be calculated from the Helmholtz free energy. Additionally, the Helmholtz free energy can be used to analyze macroscopic behaviors of materials such as phase transitions, heat capacity, and magnetic properties.

According to Zhao’s theory [20], in order to facilitate the study of the stress state of magnetic hydrogels under magnetic fields, the Helmholtz free energy is divided into an elastic part ${\tilde{W}}^{\mathrm{elastic}}(F)$ and a magnetic part ${\tilde{W}}^{\mathrm{magnetic}}(F,\tilde{B},B^{\mathrm{applied}})$. These are functions of the deformation gradient F, as well as the initial magnetic flux density $\tilde{B}$ of the material itself and the externally applied magnetic flux density $B^{\mathrm{applied}}$. The relationship between the material’s own magnetic flux density B and the strength of the external magnetic field H is given by the equation:

(12) $$B=\mu_{0}H^{\mathrm{applie}}+B_{r}$$

in which $B^{r}$ is the residual magnetic flux density of the material, $\mu_{0}$ is the magnetic permeability of the material, and $H=B^{\mathrm{applied}}/\mu_{0}$ is the external magnetic field strength. According to the characteristics of hard magnetic materials introduced in Section 2.1, due to the strong coercivity $H_{c}$ of hard magnetic materials, their strong residual magnetic flux density $B^{r}$, and low magnetic permeability *μ*, the magnitude of their magnetic flux density B will not be affected by weak external magnetic fields and will remain unchanged at the magnitude of $B^{r}$. However, because magnetic hydrogel materials are soft and can undergo significant deformation under the influence of magnetic forces, their magnetic flux density B will change with the deformation of the material, which is similar to a magnetostrictive effect. In Zhao’s theory, the magnetic part of the magnetic potential energy or Helmholtz free energy per unit volume in the current configuration, $W^{\mathrm{magnetic}}$, is defined as the work required to reorient the magnetic moments, and the expression for $W^{\mathrm{magnetic}}$ can be derived as follows:

(13) $$W^{\mathrm{magnetic}}=-\frac{1}{\mu_{0}}B\cdot B^{\mathrm{applied}}$$

Since in the reference configuration ${\tilde{W}}^{\mathrm{magnetic}}=W^{\mathrm{magnetic}}J$, according to Equation (8), the expression for the magnetic part of the Helmholtz free energy in the reference configuration is as follows:

(14) $${\tilde{W}}^{\mathrm{magnetic}}=-\frac{1}{\mu_{0}}F{\tilde{B}}^{r}\cdot B^{\mathrm{applied}}$$

In practice, the magnetic part of the Cauchy stress matrix $\sigma^{\mathrm{magnetic}}$ derived from the work conjugate relationship of the magnetic part of the free energy shows that even when B and $B^{\mathrm{applied}}$ have the same direction, the material still undergoes tensile or compressive deformation. This is physically inconsistent with Zhao’s definition of the magnetic part of the Helmholtz free energy $W^{\mathrm{magnetic}}$ as the work required to align the direction of B with $B^{\mathrm{applied}}$. Therefore, this paper redefines the magnetic part of the Helmholtz free energy $W^{\mathrm{magnetic}}$ as a magnetic potential energy, which includes two parts: one is the work required to align the direction of B with $B^{\mathrm{applied}}$, and the other is the change in magnetic potential energy due to the variation in the intensity of the material’s magnetic induction B affected by the deformation of the material. After this redefinition, it is possible to explain the phenomenon that objects will undergo tensile or compressive deformation when B and $B^{\mathrm{applied}}$ have the same direction. This is due to the occurrence of a magnetostrictive-like effect, which causes changes in the modulus of B, leading to changes in the magnetic part of the Helmholtz free energy $W^{\mathrm{magnetic}}$. At the same time, this paper defines the magnetic potential energy to be zero when the material’s magnetic induction B is perpendicular to the external magnetic induction $B^{\mathrm{applied}}$. By using Equation (11) for the magnetic flux density in the current configuration, which includes the correction factor B, to replace Equation (8) in Zhao’s expression, this paper represents the magnetic part of the Helmholtz free energy as follows:

(15) $$W^{\mathrm{magnetic}}=-\frac{1}{\mu_{0}}B\cdot B^{\mathrm{applied}}$$

The expression for ${\tilde{W}}^{\mathrm{magnetic}}$ in the reference configuration is given as follows:

(16) $${\tilde{W}}^{\mathrm{magnetic}}=-\frac{1}{\mu_{0}}(\frac{1-\alpha }{\lambda }+\alpha )F\tilde{B}\cdot B^{\mathrm{applied}}$$

This equation indicates that the magnetic part of the Helmholtz free energy ${\tilde{W}}^{\mathrm{elastic}}$ is affected by the positional changes of the object. Considering the elastic part of the Helmholtz free energy, the total Helmholtz free energy per unit volume for an ideal hard magnetic soft material in the reference configuration can be expressed as follows:

(17) $$\tilde{W}={\tilde{W}}^{\mathrm{elastic}}(F)-\frac{1}{\mu_{0}}(\frac{1-\alpha }{\lambda }+\alpha )F{\tilde{B}}^{r}\cdot B^{\mathrm{applied}}$$

Since the residual magnetic flux density ${\tilde{B}}^{r}$ in the reference configuration is a fixed vector and $B^{\mathrm{applied}}$ is independent of material properties, it is an externally controllable variable. Therefore, it can be observed that the Helmholtz free energy of the material is a function of a single variable F in this case. As the hard magnetic particle hydrogel is a typical hyperelastic material, the neo-Hookean constitutive model can be used to characterize the elastic part of its Helmholtz free energy, denoted as ${\tilde{W}}^{\mathrm{elastic}}$; thus, we have the following:

(18) $${\tilde{W}}^{\mathrm{elastic}}=\frac{G}{2}(J^{-2/3}I_{1}-3)+\frac{K}{2}{\left(J-1\right)}^{2}$$

in which G is the shear modulus of the material, K is the bulk modulus of the material, and $I_{1}=\mathrm{tr}(F^{T}F)$ is the deformation gradient. According to the principle of work conjugation:

(19) $$P=\frac{\partial \tilde{W}(F)}{\partial F}$$

According to Equation (6), the expression for the Cauchy stress can be transformed from Equation (19) as follows:

(20) $$\sigma =\frac{1}{J}\frac{\partial \tilde{W}(F)}{\partial F}F^{T}$$

Combining Equations (17)–(19), the elastic part and the magnetic part of the nominal stress can be expressed as follows:

(21) $$P^{\mathrm{elastic}}=GJ^{-2/3}(F-\frac{I_{1}}{3}F^{-T})+KJ(J-1)F^{-T}$$

(22) $$P^{\mathrm{magnetic}}=-\frac{1}{\mu_{0}}(\frac{1-\alpha }{\lambda }+\alpha )B^{\mathrm{applied}}⊗{\tilde{B}}^{r}$$

The elastic part and the magnetic part of the Cauchy stress can be obtained by transforming Equations (21) and (22) using Equation (6):

(23) $$\sigma^{\mathrm{elastic}}=GJ^{-5/3}(FF^{T}-\frac{I_{1}}{3}I)+K(J-1)I$$

(24) $$\sigma^{\mathrm{magnetic}}=-\frac{1}{\mu_{0}J}(\frac{1-\alpha }{\lambda }+\alpha )B^{\mathrm{applied}}⊗F{\tilde{B}}^{r}=-\frac{1}{\mu_{0}}(\frac{1-\alpha }{\lambda }+\alpha )B^{\mathrm{applied}}⊗FB^{r}$$

The total Cauchy stress can be expressed by combining Equations (23) and (24) as follows:

(25) $$\sigma =GJ^{-5/3}(FF^{T}-\frac{I_{1}}{3}I)+K(J-1)I-\frac{1}{\mu_{0}}(\frac{1-\alpha }{\lambda }+\alpha )B^{\mathrm{applied}}⊗FB^{r}$$

It can be seen from Equation (24) that $\sigma^{\mathrm{magnetic}}$ is a non-symmetric matrix, which results in the shear stress on the micro-element not being equal in opposite directions. The impact of this will be discussed in the following section.

### 2.3. Dynamic Equilibrium Differential Equations and Its Weak Form

**Figure 5:**
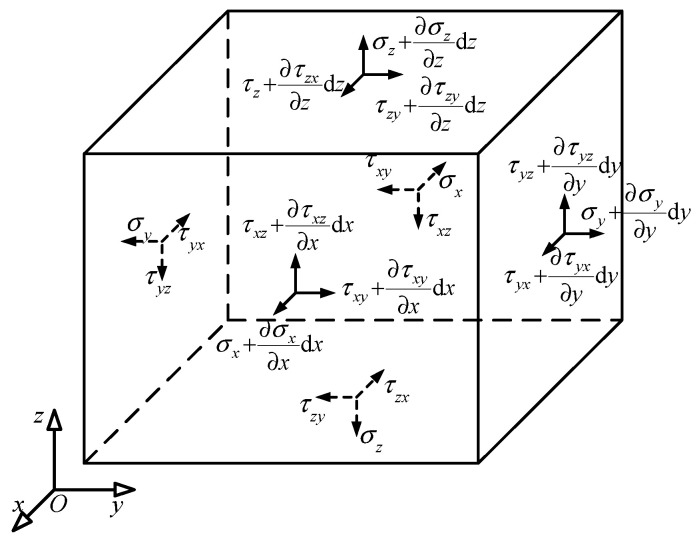
Micro-element of equivalent continuum body.

As shown in Figure 5, assume that a cubic micro-element is taken from the magnetic particle hydrogel, with dimensions d*x*, d*y*, and d*z* in the *x*, *y*, and *z* directions, respectively. The micro-element is subjected to normal stress σ and shear stress τ. According to Newton’s second law, the dynamic equilibrium equation for the equivalent continuous micro-element can be established as follows:

(26) $$\left\{\begin{array}{c} \frac{\partial \sigma_{x}}{\partial x}dxdydz+\frac{\partial \tau_{yx}}{\partial y}dydzdx+\frac{\partial \tau_{zx}}{\partial z}dzdxdy=\rho dxdydz\ddot{u}-f_{x}dxdydz\\ \begin{array}{l} \frac{\partial \tau_{xy}}{\partial x}dxdydz+\frac{\partial \sigma_{y}}{\partial y}dydzdz+\frac{\partial \tau_{zy}}{\partial z}dzdxdy=\rho dxdydz\ddot{v}-f_{y}dxdydz\\ \frac{\partial \tau_{xz}}{\partial x}dxdydz+\frac{\partial \tau_{yz}}{\partial y}dydzdz+\frac{\partial \sigma_{z}}{\partial z}dzdxdy=\rho dxdydz\ddot{w}-f_{z}dxdydz \end{array} \end{array}\right.$$

in which *u*, *v*, and *w* are the displacements of the micro-element in the *x*, *y*, and *z* directions, respectively, and *fx*, *fy*, and *fz* represent the body forces per unit volume in the *x*, *y*, and *z* directions, respectively. By dividing both sides by d*x*d*y*d*z*, the dynamic equilibrium equation in tensor form for the hydrogel equivalent continuum can be obtained as follows:

(27) $$\sigma_{ij,i}=\rho {\ddot{u}}_{j}-f_{j}$$

Due to the existence of shear stress in the magnetic part of the Cauchy stress that is not equal in opposite directions, the balance of the micro-element also needs to consider its angular momentum balance. According to the even stress elasticity theory, the angular momentum balance differential equation is as follows:

(28) $$\mu_{ji,j}+e_{ist}\sigma_{st}+M_{i}=0$$

where μ is the even stress tensor, which is the force couple generated between the contact surfaces of the micro-element and the micro-element, $e_{ist}$ is the alternating symbol, and M is the body force couple tensor. Since the model does not consider the influence of the torsion tensor, and if the volume of the hydrogel occupied by each magnetic particle on average is taken as the volume of the micro-element, its size often exceeds the size that needs to consider the even stress. Therefore, it can be considered that μ = 0, that is, the force couple produced by the asymmetric part of the Cauchy stress and the body force couple produced by the magnetic force are automatically balanced, and the virtual work problem of the micro-element rotation is no longer considered.

For the problem of magnetic hydrogel deformation under the action of a magnetic field, because it involves large deformation and the coupling of magnetic solids (magnetic field and displacement field), nonlinear problems (material and geometric nonlinearity) and unbalanced physical processes (the speed difference of the two physical fields is too large) are easily generated during the calculation process. This can lead to computational results that do not converge or have large errors when using the dynamic equilibrium differential equation as the material’s constitutive model for simulation calculations. Therefore, this paper needs to use the weak form of the differential equation as the constitutive model for simulation calculations. The weak form of the balance equation has the advantage of better handling nonlinear effects and explicitly representing coupling terms in large deformation and multi-field coupling problems.

Since the weak form of the balance differential equation is actually equivalent to the virtual work equation (also known as the Galerkin variational equation) in elasticity mechanics, the weak form of the balance differential equation can be directly used as the Galerkin variational equation for calculation as follows:

(29) $$\int \left[(\frac{\partial \sigma_{x}}{\partial x}+\frac{\partial \tau_{yx}}{\partial y}+\frac{\partial \tau_{zx}}{\partial y}+f_{x})\delta u+(\frac{\partial \tau_{xy}}{\partial x}+\frac{\partial \sigma_{y}}{\partial y}+\frac{\partial \tau_{zy}}{\partial z}+f_{y})\delta v+(\frac{\partial \tau_{xz}}{\partial x}+\frac{\partial \tau_{yz}}{\partial y}+\frac{\partial \sigma_{z}}{\partial z}+f_{z})\delta w\right]dV=0$$

### 2.4. Implementation of Numerical Computation

Through the derivation of the previous two sections, this paper has established the partial differential stress balance equation (PDE) for the magnetic hydrogel material under the condition of magneto-solid coupling, taking the displacement field u and magnetic field variables as independent variables, as its constitutive model, and has transformed this equation into the weak form of the Galerkin variational equation. This equation can describe the problem of the magnetic hydrogel material generating motion and deformation under the stimulation of the magnetic field. Since partial differential equations often cannot obtain analytical solutions, the finite element method is needed to solve this problem numerically.

Comsol, as a finite element analysis software, has convenient multi-physics coupling functions and the flexibility to insert custom weak form partial differential equations at will. Therefore, this paper chooses Comsol finite element analysis software to perform numerical calculations on the problem of magnetic hydrogel soft robots responding under magnetic field stimulation. Considering that the derived constitutive equation mainly takes the displacement field u as the independent variable, and the external magnetic field $B^{\mathrm{applied}}$ is controllable by humans, the calculation uses Comsol’s solid mechanics module, where the custom weak form partial differential function is used to insert the derived constitutive model. The general solution equation built into Comsol is

(30) $$\int_{\Omega }weak\partial s=0$$

where weak is the weak term within the solution domain Ω. Since Comsol has built-in gravity and neo-Hookean constitutive models and their weak forms, we can ignore the Cauchy stress elastic part $\sigma^{\mathrm{elastic}}$ and the body force f when setting the custom weak form partial differential equation, and only need to define the Cauchy stress elastic part $\sigma^{\mathrm{magnetic}}$.

(31) $$weak=-{\sigma_{ij}}^{\mathrm{magnetic}}\frac{\delta u}{\delta x_{i}}$$

## 3. Numerical Examples for Validation

To verify the correctness and feasibility of the numerical calculation method in this paper, this section carries out simulation calculations on numerical example 1 with analytical solutions: the uniaxial tension and compression problem of the magnetic hydrogel material cube, and numerical example 2 with experimental data: the bending of the cantilever beam under the magnetic field, comparing the numerical results with experimental data and analytical solutions for verification.

### 3.1. Example 1: Uniaxial Extension and Compression of a Cubic

**Figure 6:**
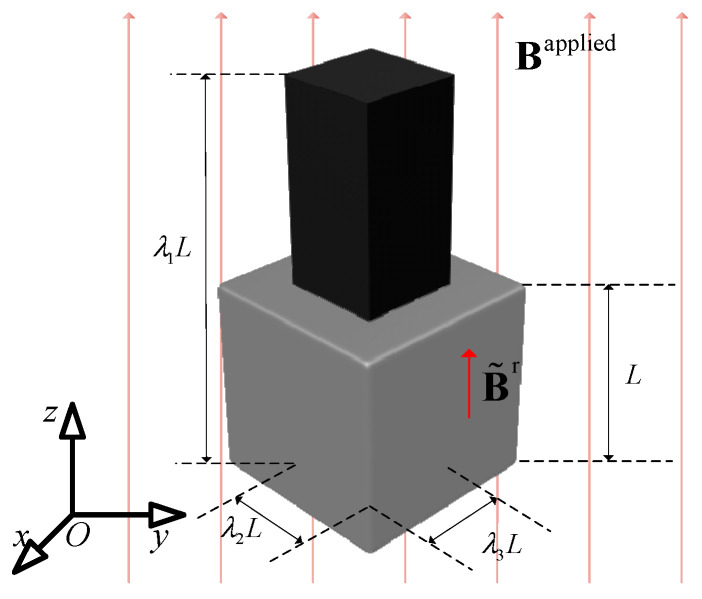
Uniaxial extension and compression of a cubic of magnetic hydrogel.

Figure 6 shows the uniaxial tension and compression model of the magnetic hydrogel cube in the magnetic field. The initial edge length of the cube is *L*, and it has a residual magnetic flux density uniformly distributed in the *z*-direction. The direction of the external magnetic field $B^{\mathrm{applied}}$ is also in the *z*-direction. The bottom boundary of the cube is set to have a *z*-direction displacement constraint; that is, the displacement component *w* of the bottom boundary is 0, and it is only allowed to slide on the o-*x-y* plane. According to the imposed conditions, the cube has no shear strain, only principal strain. Let the principal stretch coefficients in the *x*, *y*, and *z* directions of the cube be *λ_x_*, *λ_y_*, and *λ_z_*, respectively. Due to the symmetry of the material and load, $\lambda_{x}=\lambda_{y}$, and the system’s deformation gradient matrix can be represented by the following formula:

(32) $$F=\left(\begin{array}{ccc} \lambda_{x} & 0 & 0\\ 0 & \lambda_{y} & 0\\ 0 & 0 & \lambda_{z} \end{array}\right)$$

Since the material is a hyperelastic material with the characteristic of incompressibility, it can be assumed that $J=\lambda_{x}\lambda_{y}\lambda_{z}=1$. Also, because $\lambda_{x}=\lambda_{y}$, $\lambda_{x}=\lambda_{y}={\lambda_{z}}^{-1/2}$, and the deformation gradient matrix can be represented as follows:

(33) $$F=\left(\begin{array}{ccc} {\lambda_{z}}^{-1/2} & 0 & 0\\ 0 & {\lambda_{z}}^{-1/2} & 0\\ 0 & 0 & \lambda_{z} \end{array}\right)$$

Let the modulus of the external magnetic field $B^{\mathrm{applied}}$ be $\left|B^{\mathrm{applied}}\right|$, and the modulus of the material’s residual magnetic flux density $B^{r}$ be $\left|B^{r}\right|$. It can be represented as follows:

(34) $$B^{\mathrm{applied}}=\left(\begin{array}{c} 0\\ 0\\ \left|B^{\mathrm{applied}}\right| \end{array}\right),\;B^{r}=\left(\begin{array}{c} 0\\ 0\\ \left|B^{r}\right| \end{array}\right)$$

Ignoring the effect of gravity, according to the Cauchy stress formula (25), it can be obtained as follows:

(35) $$\sigma =\left(\begin{array}{ccc} \frac{G}{3}(\lambda_{z}^{-1}-\lambda_{z}^{2}) & 0 & 0\\ 0 & \frac{G}{3}(\lambda_{z}^{-1}-\lambda_{z}^{2}) & 0\\ 0 & 0 & \frac{2G}{3}(\lambda_{z}^{2}-\lambda_{z}^{-1})-\frac{\lambda_{z}}{\mu_{0}}(\frac{1-\alpha }{\lambda_{z}}+\alpha )\left|B^{r}\right|\left|B^{\mathrm{applied}}\right| \end{array}\right)$$

Due to the existence of free boundaries, it is necessary to have the following:

(36) $$\sigma_{x}=\sigma_{y}=\sigma_{z}=0$$

Combining the boundary conditions with Formula (35), the dimensionless quantity equation can be obtained as follows:

(37) $$\frac{\lambda_{z}^{2}-\lambda_{z}^{-1}}{1-\alpha +\lambda_{z}\alpha }=\frac{\left|B^{r}\right|\left|B^{\mathrm{applied}}\right|}{\mu_{0}G}$$

**Figure 7:**
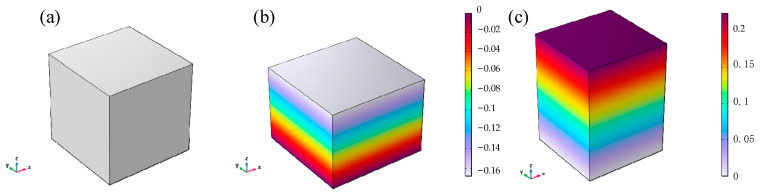
Numerical solutions for displacement contour of the magnetic hydrogel cube in the *z* direction. (**a**) Initial undeformed state with no external magnetic field; (**b**) compressed deformation under the action of an external magnetic field (*λ_z_* = 0.83); (**c**) extension state under the action of an external magnetic field (*λ_z_* = 1.22).

For the cube model with the same shear modulus G and residual magnetic flux density $B^{r}$, different external magnetic flux densities $B^{\mathrm{applied}}$ are applied. The numerical results and analytical solutions of the *z*-direction principal elongation ratio are compared. The displacement cloud diagram of the numerical results is shown in Figure 7.

**Figure 8:**
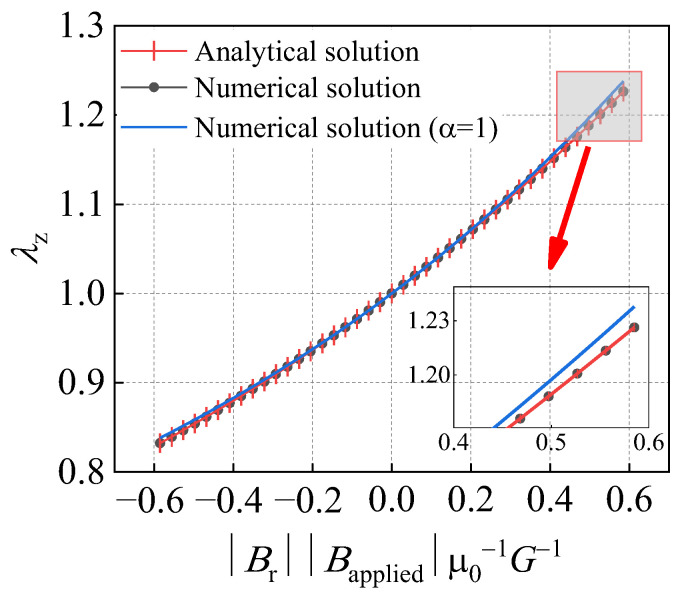
Uniaxial extension and compression of the magnetic hydrogel cube in the magnetic field.

The comparison curve between the simulation result data and the theoretical solution, and the correction coefficient α is 1 (i.e., the effect of the magnetic flux direction magnetic domain density is not considered), as shown in Figure 8. From Figure 8, it can be seen that the simulation results are completely consistent with the equation calculation results. The simulation results without considering the correction coefficient α will gradually produce errors with the increase in the absolute value of the elongation ratio when considering the correction coefficient α.

### 3.2. Example 2: Bending of Cantilever Beam Under Magnetic Field

Similarly, to verify the correctness and feasibility of the numerical calculation method in this paper, and to provide a basis for the subsequent simulation work of the motion of magnetic hydrogel soft robots under magnetic field stimulation, this paper carries out numerical simulation on the bending experiment of the magnetic hydrogel cantilever beam mentioned in the previous text, and compares the numerical results with the experimental results and theoretical values to verify the correctness of the numerical simulation.

(1) Theoretical solution of the deflection of the cantilever beam

Refer to the settings and related material parameters in Section 2.2, and set the cantilever beam as a rectangular beam with dimensions of 18 mm × 4.9 mm × 1.76 mm (*L* × *W* × *C*). Set its residual magnetic flux density to 22.5 mT, and the magnetization direction is upward in the thickness direction. The left boundary is set as a fully constrained fixed boundary, and the remaining boundaries are free boundaries. To obtain the analytical solution of the deflection of this cantilever beam bending problem, it can be assumed that under small deflection, the *z*-coordinate of each point along the *L* direction remains unchanged. It can be further considered that under the action of the magnetic field, it consistently maintains the reference configuration without change; that is, the deformation gradient matrix is always

(38) $$F=\left(\begin{array}{ccc} F_{xx} & 0 & 0\\ 0 & F_{yy} & 0\\ 0 & 0 & F_{zz} \end{array}\right)=\left(\begin{array}{ccc} 1 & 0 & 0\\ 0 & 1 & 0\\ 0 & 0 & 1 \end{array}\right)$$

At this time, for the Cauchy stress magnetic part $\sigma^{\mathrm{magnetic}}$, the only non-zero element is

(39) $$\sigma_{zy}^{\mathrm{magnetic}}=-\mu_{0}^{-1}\left|B^{\mathrm{applied}}\right|\left|{\tilde{B}}^{r}\right|$$

**Figure 9:**
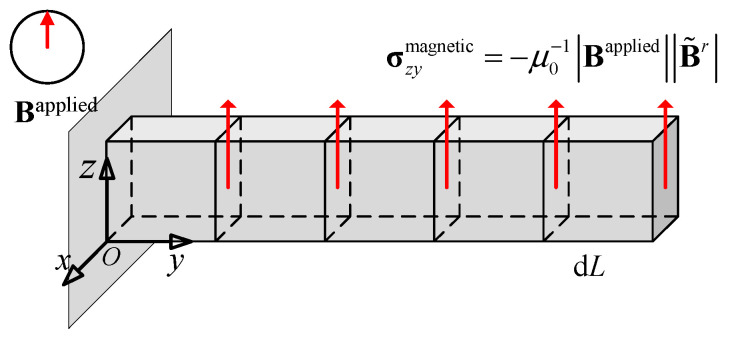
Schematic diagram of the magnetic hydrogel cantilever beam under the magnetic field force.

As shown in Figure 9, if the cantilever beam is decomposed into micro-element bodies with a length of d*L* along the axial direction, it can be considered that each micro-element body is subjected to a force couple generated by the unequal shear stress part $\sigma_{yz}^{\mathrm{magnetic}}$. From this, the bending moment on the beam section at position y can be calculated as follows:

(40) $$M(x)=\mu_{0}^{-1}\left|{\tilde{B}}^{r}\right|\left|B^{\mathrm{applied}}\right|WC(L-y)$$

If the total bending moment is regarded as an equivalent bending moment produced by a point load acting on the right free end, the size of this point load can be represented by the following formula:

(41) $$Q=\left|dM/dx\right|=\mu_{0}^{-1}\left|{\tilde{B}}^{r}\right|\left|B^{\mathrm{applied}}\right|WC$$

The deflection can then be expressed as follows:

(42) $$\delta_{\max}=\frac{4\left|{\tilde{B}}^{r}\right|\left|B^{\mathrm{applied}}\right|L^{3}}{3\mu_{0}GC^{2}}$$

**Figure 10:**
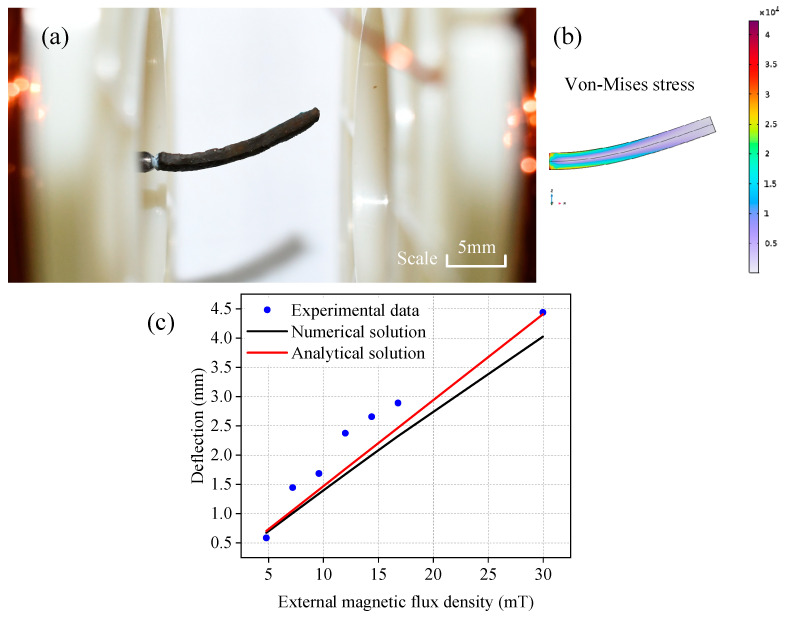
Bending of the hydrogel cantilever beam: (**a**) deflection of the magnetic polyvinyl alcohol (PVA) hydrogel cantilever beam in the experiment under 30 mT; (**b**) deflection of the magnetic PVA hydrogel cantilever beam in the simulation under 30 mT; (**c**) comparison of experimental, theoretical, and simulation deflection results of the magnetic PVA cantilever beam under different external magnetic induction intensities.

Similarly, using the material parameters obtained from the experiment, set the shear modulus *G* = 306 Kpa, and set the material as an incompressible hyperelastic body. The calculation results using formula (42) are compared with the numerical simulation results and the experimental results to obtain the curve in Figure 10. Figure 10a,b show the bending results of the magnetic hydrogel cantilever beam obtained from the experiment and simulation under a 30 mT external magnetic induction intensity load, respectively.

(2) Materials and methods for the bending experiment of the cantilever beam

This study utilizes a cyclic freezing–thawing physical crosslinking technique to fabricate polyvinyl alcohol (PVA) hydrogels. Throughout the fabrication process, a mixture containing neodymium–iron–boron (NdFeB) magnetic particles and PVA hydrogel is sonicated in an ultrasonic bath to eliminate bubbles. The resulting magnetic PVA hydrogel specimens exhibit a water content of 62.5%, a PVA concentration of 12.5%, and incorporate 2000-mesh NdFeB magnetic powder (with particle diameters ranging from 6 to 7 μm) at a proportion of 28.6%.

For the mechanical property test of the PVA hydrogel materials, specimens were crafted into standard dumbbell-shaped test pieces with precise dimensions: an overall length of 115 mm, end widths of 25 mm, a narrowed middle width of 6 mm, a narrowed length of 33 mm, and a thickness of 2 mm. In the cantilever beam bending tests, the beams were configured as rectangular sections with dimensions of 18 mm × 4.9 mm × 1.76 mm (length × width × height). We initiated the process by applying pulse magnetization to the thickness direction (*z*-axis) of the magnetic PVA hydrogel. Post-magnetization, the residual magnetic flux density of the magnetic PVA hydrogel was measured using a handheld Tesla meter, yielding a value of 22.5 mT. The hydrogel was then positioned with one end fixed and the other end free in an external magnetic field aligned parallel to its length, ensuring that the external magnetic field was perpendicular to the material’s residual magnetic field. By varying the external magnetic field strength and monitoring the deflection at the free end, we investigated the response of the magnetic PVA hydrogel to different magnetic field intensities. A custom-built Helmholtz coil was employed to impose an external magnetic field load on the magnetic PVA hydrogel cantilever beam, with the magnetic flux density being determined by a Tesla meter. By modulating the current through the direct current power supply of the Helmholtz coil, we applied varying magnetic flux density loads to the cantilever beam. The experiments revealed that under magnetic flux density loads of 4.8 mT, 7.2 mT, 9.6 mT, 12 mT, 14.4 mT, 16.8 mT, and 30 mT, the deflections of the magnetic hydrogel cantilever beam were 0.58 mm, 1.44 mm, 1.69 mm, 2.37 mm, 2.65 mm, 2.89 mm, and 4.44 mm, respectively.

As shown in Figure 10c, it can be seen that the gap between the numerical simulation data and the theoretical solution increases with the increase in deflection, but does not exceed ten percent. Although the experimental data generally agrees with the overall trend of the numerical simulation data and the theoretical solution, there are large fluctuations. This paper believes that this is because the hydrogel samples prepared in this paper need to be close to the ferromagnetic medium during the magnetization process, and the ferromagnetic medium often generates a strong mutual attraction at the moment of magnetization. The magnetic hydrogel samples are squeezed by the attraction of the surrounding ferromagnetic media, causing the shape of the samples to change to a certain extent. In addition, since the magnetic PVA hydrogel material manufactured in this paper has a strong nonlinearity and has different shear moduli at different deformation stages, there will be a certain error between the shear modulus set in the numerical simulation and the actual situation. According to the comparison between the numerical simulation data and the theoretical and experimental data, excluding the experimental data points with large fluctuations, the gap between the simulation results and the theoretical solution does not exceed 10%, and it is generally consistent with the overall trend of the experimental data. The error is smaller when the deformation is larger, and the error is 10% at 30 mT, which can be considered to have correctness. Two experimental shortcomings may be the reasons for the errors in the results: first, the shape and dimensions of the hydrogel have undergone some changes during magnetization. Second, the distribution of magnetic particles within the hydrogel is not 100% perfectly uniform.

## 4. Application to the Design of Magnetically Actuated Hydrogel Soft Robots

### 4.1. Structural and Load Preliminary Design

**Figure 11:**
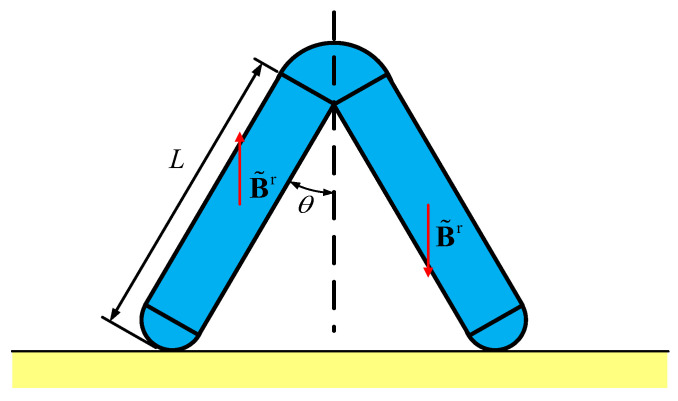
Schematic diagram of the double-feet magnetic hydrogel soft robot.

As shown in Figure 11, the magnetic gel soft robot designed in this paper has a zigzag structure, which can be considered as a bipedal structure with a certain angle 2θ. The residual magnetic flux density of the two feet is in opposite directions, pointing upwards and downwards. It relies on gravity, friction, and magnetic force to achieve the lifting and bending of the two feet, thereby achieving inchworm-style bipedal walking motion.

The reason for adopting the zigzag structure is based on the need for stability, flexibility, and imitation of biological motion. The zigzag structure can provide better stability and balance. In addition, the flexibility and adaptability of the zigzag structure enable the robot to adapt to various environments and tasks. By adjusting the position, angle, and motion mode of the front and rear feet, the robot can overcome obstacles and move in narrow, irregular, or complex environments. At the same time, the zigzag structure can also imitate the motion mode of the inchworm, thereby achieving the robot’s advancement. Compared with other complex structures, the control and programming of the zigzag structure are also relatively simple.

The material parameters required are obtained from the mechanical property test of the PVA hydrogel materials, as shown in Table 1. The coefficient of friction cannot be accurately determined by traditional experimental methods due to the adsorption effect between the hydrogel and the contact surface, so the coefficient of friction is roughly selected as 0.4 when using the Coulomb friction model for calculation.

**Figure 12:**
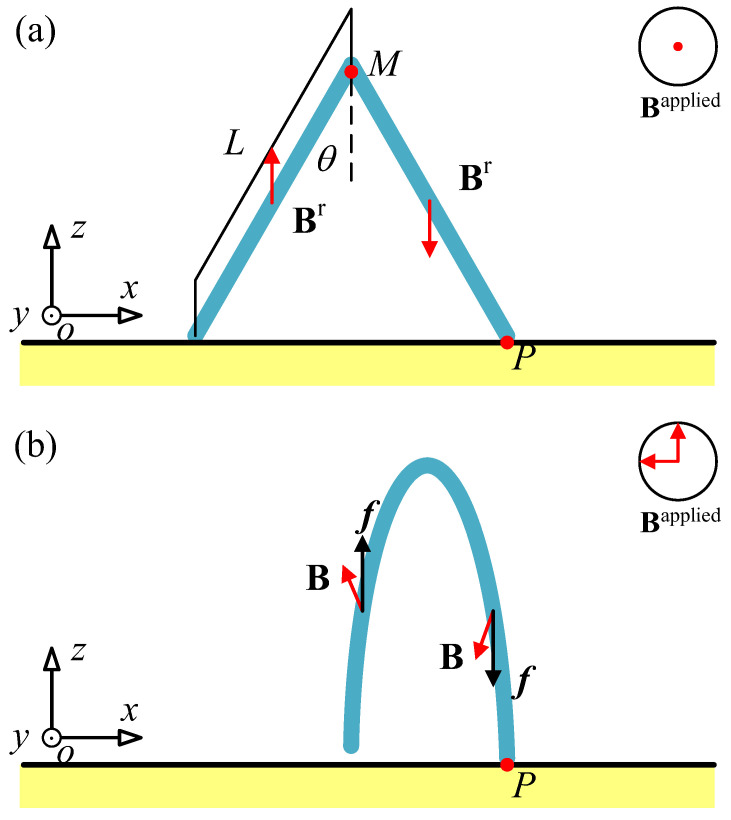
Deformation and force of the designed bipedal magnetic hydrogel soft robot under the magnetic field. (**a**) The initial configuration of the soft robot, L is the foot length, is half of the foot angle, **B**^r^ is the residual magnetic flux density of the material, **B** is the magnetic flux density of the material in the instantaneous configuration, $B^{\mathrm{applied}}$ is the external magnetic field strength applied, M is the center point of the structure, P is the contact point; (**b**) under the external magnetic field strength in the x-direction, the bipedal structure bends inward, and at this time, due to the different bending deflections of each point on the foot length, each point B is also different, thus generating different directions of forces on the two feet under the external magnetic field strength in the *z*-direction, and the rear foot lifts off the contact surface.

The motion mode of the hydrogel soft robot designed in this paper is an inchworm-style bipedal walking motion. The specific motion can be divided into four stages. Stage one: slowly apply a magnetic field along the *z*-axis. Due to the opposite magnetization directions of the front and rear feet, during the deformation process of the two feet, the front leg will be subjected to a downward force and the rear leg will be subjected to an upward force. This will cause the overall structure to rotate a small angle around the support point of the front leg, allowing the rear leg to lift off the contact surface. Stage two: after the rear leg leaves the contact surface, apply a magnetic field in the *x*-direction. Under the action of the magnetic field, the bipedal structure will bend inward. Since the rear leg is not subjected to friction force after leaving the contact surface, the center of gravity of the overall structure will move towards the support point of the front leg during the bending process. At the same time, slowly withdraw the magnetic field in the *z*-direction during the bending process, so that after the bending is stable. The rear leg will come into contact with the contact surface again. Stage three: similar to the first step, apply a magnetic field in the opposite direction to the *z*-axis, allowing the front leg to lift off the contact surface. Stage four: slowly withdraw the magnetic field in the *x*-direction and the *z*-direction; the bending of the bipedal structure will gradually disappear. Since the front leg is not subjected to friction force after leaving the contact surface, the center of gravity of the overall structure will move forward during the disappearance of the bending. After the magnetic field completely disappears, the front leg will come into contact with the contact surface again, and the structural configuration will return to the original undeformed state. These four stages of motion constitute a cycle of the inchworm-style motion of the hydrogel soft robot, and the specific motion is shown in Figure 12.

The above motion process depends on the comprehensive action of gravity, magnetic force, and friction force. By controlling the magnitude and direction of the external magnetic field $B^{\mathrm{applied}}$, the motion process can be controlled. The magnitude and direction of $B^{\mathrm{applied}}$ directly determine whether the motion can be achieved and the efficiency of the advance. In order to calculate the appropriate external magnetic field $B^{\mathrm{applied}}$ as the load, the model of the hydrogel soft robot is first simplified to the two fixed-length rods shown in Figure 12. Under the influence of the applied external magnetic field strength $B^{\mathrm{applied}}$, the structure deforms as shown in Figure 12b. During the deformation process, the magnetic field strength **B** of every point of the soft robot itself will change with the deformation of the structure. Since the magnetic field strength in the *x*-direction is the same at every moment, and in the *z*-direction is the opposite, in the simplified model, one side of the rod is subjected to a downward force, and the other side of the rod is subjected to an upward force. At the same time, the forces on the two rods in the *x*-direction are also equal in magnitude and opposite in direction. At this time, under the condition of overall force balance, a torque is generated at point P, causing the rod subjected to the upward force to lift slightly. The requirements for the applied $B^{\mathrm{applied}}$ are to lift the rod to a small height; that is, to balance the torque of gravity at point P, and at the same time, to try to keep point P from sliding.

The forces in opposite directions generated on the two rods can be explained by the dynamic equilibrium differential Equation (26). When only considering the magnetic field strength *B*_z_ and $B_{z}^{\mathrm{applied}}$ in the z-direction of the material and the external environment, since the deformation is symmetrical, the Cauchy stress elastic part $\sigma^{\mathrm{elasitc}}$, inertial force, and gravity in the same direction and equal magnitude on the two rods are ignored. At this time, the Cauchy stress only has the magnetic part of the z-component $\sigma_{z}^{\mathrm{magnetic}}$ as a non-zero element. Equation (26) can be simplified to Equation (43) as follows:

(43) $$\frac{\partial \sigma_{z}}{\partial z}=-f_{z}$$

where *f_z_* is the body force in the *z*-direction. Since every point in the initial configuration has the same $\sigma_{z}^{\mathrm{magnetic}}$, that is, $\partial \sigma_{z}/\partial z$ is 0, then there will be no force generated. During the bending process of the two feet, $\partial \sigma_{z}/\partial z$ will not be 0 due to the different deformation gradients at each point, setting the vertical distance of each point on the rod from point P as *x*, the torque balance equation for point P can be expressed as follows:

(44) $$\int_{0}^{2L\sin\theta }\rho gxdx=\int_{L\sin\theta }^{2L\sin\theta }\frac{\partial \sigma_{z}^{\mathrm{magnetic}}}{\partial z}xdx-\int_{0}^{L\sin\theta }\frac{\partial \sigma_{z}^{\mathrm{magnetic}}}{\partial z}xdx$$

Since the specific structure is not a simple rod, and it involves large deformation problems, it is necessary to test and obtain reasonable $B_{z}^{\mathrm{applied}}$ in the numerical simulation process to make the soft robot complete the first and third parts of the designed motion in the previous text. In addition, it is found in the simulation process that if a pulse load is applied, it will affect the overall stability of the structure’s motion. Therefore, this paper chooses to apply a triangular wave form of external magnetic field strength instead of a pulse wave, and smooth processing is performed. After multiple numerical calculations and optimization, the peak value of this triangular wave is finally set to 15 mT.

For the second part of the motion, the bipedal structure bends towards the centerline of the structure under the action of the *x*-direction magnetic field $B_{x}^{\mathrm{applied}}$, thus moving the center of gravity forward. On the premise that the lift of the rear leg after the first step is very small, it can be considered that the height of the front and rear feet is the same. Then, the *x*-direction magnetic field $B_{x}^{\mathrm{applied}}$ makes the front and rear feet obtain a load that is the same in size and opposite in direction, which can be considered as the load always being in a balanced state. Therefore, the magnetic field $B_{x}^{\mathrm{applied}}$ should make the bending deflection of the two feet as large as possible, so that the step size of the magnetic hydrogel soft robot can be as large as possible under the structural restrictions, thereby improving the efficiency of motion. The *z*-direction magnetic field load $B_{z}^{\mathrm{applied}}$ in the third part of the motion can refer to the magnetic field size of the first step, and the fourth part of the motion only needs to slowly decay the magnetic field load to 0.

To verify the rationality of the designed motion, this paper has conducted numerical simulation on the case where the bipedal angle 2*θ* = 60° (the foot length *L* and foot width and thickness refer to the settings in Section 4.2) to achieve its motion. After calculation and optimization, $B_{z}^{\mathrm{applied}}$ is applied with a triangular wave peak of 15 mT, and then a periodic sawtooth wave is applied (its peak value is temporarily set to 80 mT) as $B_{x}^{\mathrm{applied}}$. Their functions change with time in two cycles (one cycle time is set to 3 s, and there is a 1 s interval between each cycle) as shown in the following figure.

**Figure 13:**
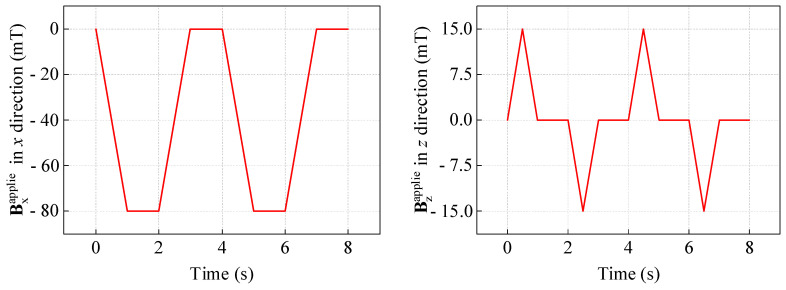
Variation of magnetic field strength loads in the *x* and *z* directions over time.

As shown in Figure 13, the *x*-direction magnetic field $B_{x}^{\mathrm{applied}}$ in one cycle rises from 0 to the peak in the first 1 s, maintains for 1 s, and then decreases back to 0 in 1 s. This enables it to achieve the inward bending and rebound of the hydrogel soft robot’s bipedal structure within one cycle. The *z*-direction magnetic field $B_{z}^{\mathrm{applied}}$ exists in the form of triangular waves with different directions and the same peak size in the first 1 s and the last 1 s of one cycle, which achieves the lifting of the front and rear feet in the first and third parts of the motion.

### 4.2. System Parameter Optimization

At present, the research on magnetic soft robots is often focused on preparing a simple soft robot structure and achieving its motion, lacking a forward design and parameter optimization process. Based on the magnetic soft robot structure and motion mode given in the previous text, this section carries out forward design and parameter optimization on the bipedal angle and the size of the x-direction magnetic flux density load applied in the second part of the motion for the bipedal magnetic hydrogel soft robot with given foot length and thickness.

A. Optimization of foot length and thickness

According to the motion mode of the hydrogel soft robot designed in this paper, its motion efficiency can be considered to depend on the stride and frequency. If, according to bionics, it is required to complete a motion cycle in the same time as the motion cycle of the inchworm, then its motion efficiency will basically depend on the size of the stride. The larger the stride, the higher the motion efficiency, and the smaller the stride, the finer the motion and the lower the motion efficiency.

**Figure 14:**
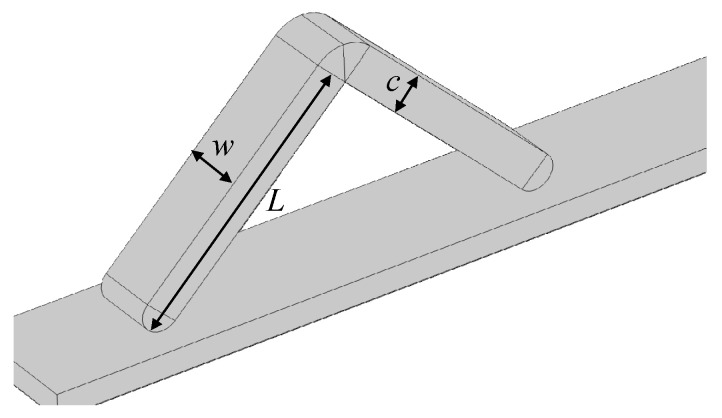
Schematic diagram of the magnetic bipedal hydrogel soft robot structure (where *W* is the width, *L* is the foot length, and *c* is the thickness).

In the previous cantilever beam experiments, we can see that under the action of the external magnetic field, the deflection of the magnetic hydrogel cantilever beam depends on the length of the cantilever beam and the size of the external magnetic field load. Therefore, under the same bipedal angle and the same *x*-direction external magnetic field strength $B_{x}^{\mathrm{applied}}$, the longer the foot length *L*, the larger the stride will be. Similarly, the larger the thickness *c*, the smaller the deflection. The parameterized simulation calculation results of the magnetic hydrogel cantilever beam thickness in the previous text show that the larger the width, the smaller the bending deflection of the magnetic hydrogel cantilever beam under the same external magnetic flux density. It can be considered that the larger the width of the soft robot, the smaller the stride will be. Therefore, there is no need for parameter optimization for length *L*, width *w*, and thickness *c* of the magnetic hydrogel soft robot structure. The size of the magnetic bipedal hydrogel soft robot in each direction is shown in Figure 14.

Therefore, in order to facilitate the parameterized optimization design of the bipedal angle 2*θ* and the *x*-direction magnetic flux density load $B_{x}^{\mathrm{applied}}$, we might as well determine the size of the foot length *L*, width *w*, and thickness *c* according to bionics, imitating the body size of the cloud inchworm larva. The body length of the mature cloud inchworm larva is 40–60 mm. Therefore, based on bionics, this paper sets the foot length of the designed hydrogel soft robot to 21.5 mm, the width to 3 mm, and the thickness to 5 mm. At the same time, its motion cycle is set to 3 s, and it is evenly distributed to the three processes of the bipedal beginning to bend, the bipedal remaining bent unchanged, and the bipedal rebounding.

B. Optimization of Biped Gait Angle and Magnetic Field Load

This paper equates the motion efficiency of the soft robot to the distance moved by the center point within one motion cycle. As described above, the foot length, width, and thickness of the hydrogel soft robot have been determined according to bionics, and the cycle time has also been determined according to the load setting. The distance that the center point of the soft robot can move within one cycle can be considered as the two strides produced by the bending and rebounding of the bipedal structure in the second and fourth stages of motion. Therefore, the stride directly determines its motion efficiency.

**Figure 15:**
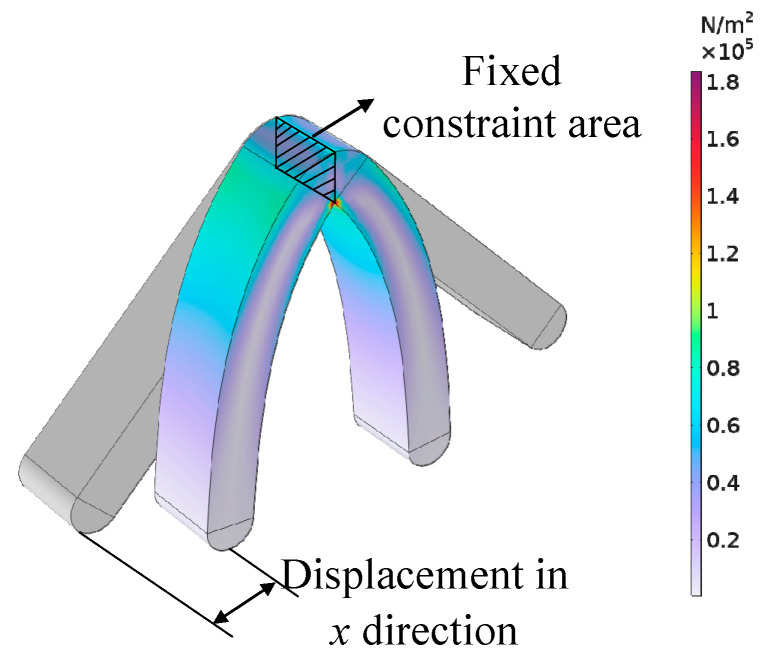
Model used for parameterized optimization.

Under the condition that the size of the soft robot is determined, the size of the stride depends on the bipedal angle of the soft robot and the magnetic field load $B_{x}^{\mathrm{applied}}$ that causes the bipedal bending. Therefore, this paper chooses to optimize the bipedal angle and the peak value of the magnetic field load $B_{x}^{\mathrm{applied}}$. Since the two feet of the designed bipedal hydrogel soft robot are completely symmetrical when bent under $B_{x}^{\mathrm{applied}}$, and they do not contact the contact surface at the same time during the bending process, this paper applies a fixed constraint to the symmetrical surface of the soft robot structure, removes the contact surface and the *z*-direction magnetic flux density load $B_{z}^{\mathrm{applied}}$, and studies the displacement of the end of the leg structure in the *x*-direction under different 1/2 bipedal angles θ and peak values of the magnetic field load $B_{x}^{\mathrm{applied}}$ to replace the study of the stride. The simulation calculation model is shown in Figure 15.

**Figure 16:**
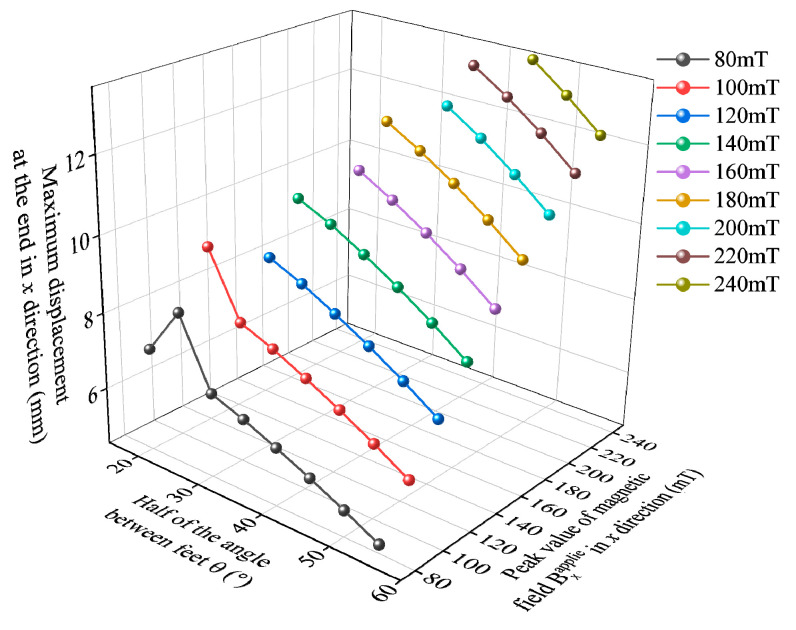
Displacement of the end of the foot structure in the *x*-direction for different peak values of magnetic field load of 80~240 mT and 1/2 bipedal angles of 20–55°.

By using Comsol for transient calculations of the deformation of different magnetic field load peak values $B_{x}^{\mathrm{applied}}$ and bipedal angles within 1.2 s, a three-dimensional scatter plot of the displacement of the end of the foot structure in the *x*-direction is obtained as shown in Figure 16. Analysis of the data shows that under the same bipedal angle, the larger the peak value of the magnetic field load $B_{x}^{\mathrm{applied}}$, the greater the bending degree of the two feet. Under the same peak value of the magnetic field load $B_{x}^{\mathrm{applied}}$, the smaller the bipedal angle, the greater the bending degree of the two feet. However, due to structural limitations, too small a bipedal angle will often cause the foot structure to bend and contact each other under a larger magnetic field load $B_{x}^{\mathrm{applied}}$, and even if the magnetic field load is increased, it will not increase the displacement of the end of the foot structure in the *x*-direction.

**Figure 17:**
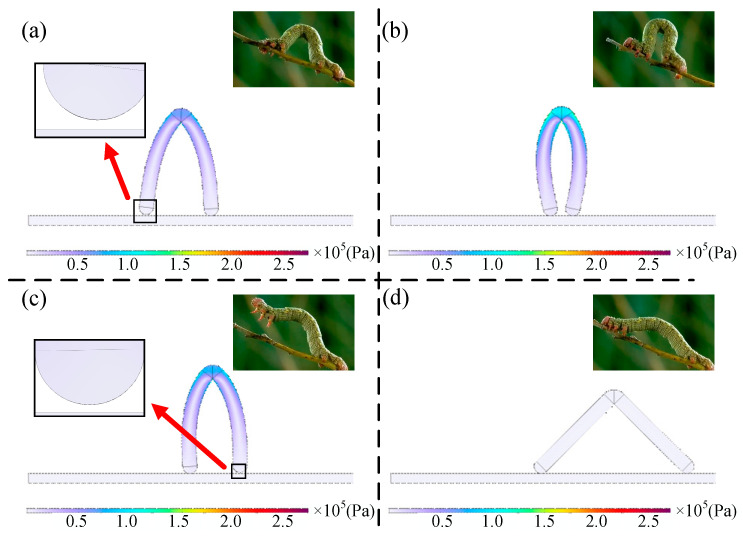
Schematic diagram of the soft robot’s motion within one cycle (Von Mises stress contour) compared with the motion of the inchworm. (**a**) The rear foot leaves the contact surface at 0.55 s; (**b**) the two feet complete the inward bending at 1.32 s; (**c**) the front foot leaves the contact surface at 2.33 s; (**d**) the motion cycle is completed.

Within the given data range, it can be shown that when the peak value of the magnetic field load $B_{x}^{\mathrm{applied}}$ is 240 mT and the 1/2 bipedal angle θ is 45°, the displacement of the end of the foot structure in the *x*-direction is the largest, reaching 13.58 mm. Under this condition, it can be considered that the soft robot can move the center point M forward by about 27.16 mm within one cycle. The motion of the designed soft robot within one cycle is shown in Figure 17 using a 1/2 bipedal angle θ of 45° and a peak value of $B_{x}^{\mathrm{applied}}$ 240 mT for calculation.

The motion states of the soft robot shown in Figure 17 at four different times fully conform to the motion process designed in Section 4.1, which includes four stages within one motion cycle. Stage one: under the influence of the magnetic induction intensity in the positive z-direction, the hind leg lifts off the contact surface, with the friction force provided entirely by the front foot. Stage two: after the hind leg leaves the contact surface, under the influence of the magnetic field in the negative *x*-direction, the dual-foot structure bends inward, and the center of gravity moves in the positive x-direction. Stage three: similar to the first step, under the influence of the magnetic induction intensity in the negative *z*-direction, the front leg lifts off the contact surface, with the friction force provided entirely by the hindfoot. Stage four: the magnetic fields in the *x* and *z* directions are removed, the bending of the dual-foot structure gradually disappears, the center of gravity moves in the positive *x*-direction, and the structural configuration returns to its original undeformed state.

**Figure 18:**
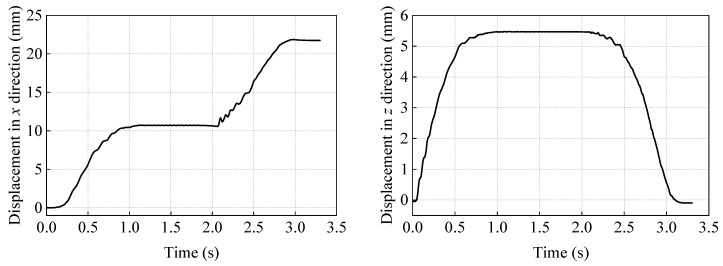
Displacement of the soft robot’s center point M in the *x* and *z* directions within one cycle.

As shown in Figure 18, the *x*-direction displacement of the soft robot’s center point M comes from the inward bending of the dual-foot structure during the first 1 s of a motion cycle and the rebound of the dual-foot structure during the last 1 s. The middle 1 s is the bending maintenance stage, so no displacement is generated. In one cycle, it moves 21.7 mm in the positive *x*-direction, and the difference from the predicted 27.16 mm in the previous text comes from the sliding of the contact point. The *y*-direction displacement of the soft robot’s center point also comes from the inward bending of the dual-foot structure, with the same trend as the *x*-direction displacement, rising during the first 1 s of the motion cycle and returning to the initial height in the last 1 s, with no change during the middle 1 s of the bending maintenance stage.

**Figure 19:**
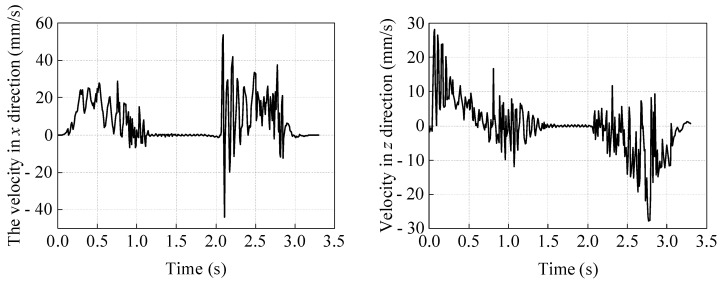
Velocity of the soft robot’s center point M in the *x* and *z* directions within one cycle.

As shown in Figure 19, the *x*-direction velocity of the soft robot’s center point quickly increases to about 20 mm/s within the first 0.3 s of a cycle, then decreases in fluctuations until it reaches zero at 1 s. At 2 s, it starts to increase again in fluctuations, reaching about 20 mm/s around 2.5 s, and then decreases to zero before 3 s in fluctuations. It should be noted that the *x*-direction velocity is accompanied by obvious fluctuations during the motion process, which is due to the sliding of the contact point between the soft robot and the contact surface, and the changes in external magnetic induction intensity loads also cause the structure to vibrate slightly. The *y*-direction velocity of the soft robot’s center point has a similar trend to the *x*-direction velocity, with the difference being that its velocity direction is opposite between the 2–3 s stage and the 0–1 s stage, and it also produces fluctuations during the motion process.

### 4.3. Influence of Coefficient of Friction on Motion

The implementation of the soft robot’s motion pattern designed in this paper relies on the combined effects of external magnetic induction intensity, gravity, and frictional force. The combination of external magnetic induction intensity in the *z*-direction and gravity is responsible for achieving the separation of the fore and aft foot structures from the contact surface at the required times. The external magnetic induction intensity in the *x*-direction is responsible for the bending of the dual-foot structure, while the presence of frictional force is responsible for maintaining the contact between the fore and aft feet with the contact surface without relative sliding during the inward bending process of the dual-foot structure. When the height at which the fore and aft foot structures are separated from the contact surface is very small, the load experienced by the soft robot during the inward bending process of the foot structures is symmetrical to the overall structure of the soft robot. Therefore, it does not generate additional body forces in the direction of structural movement. Theoretically, as long as the inertial force of the structure does not exceed the critical friction force, the foot structures in contact with the contact surface will not produce relative sliding. However, due to the vibration of the structure under the influence of external magnetic induction, this friction force often needs to be greater than the inertial force to maintain non-sliding.

**Figure 20:**
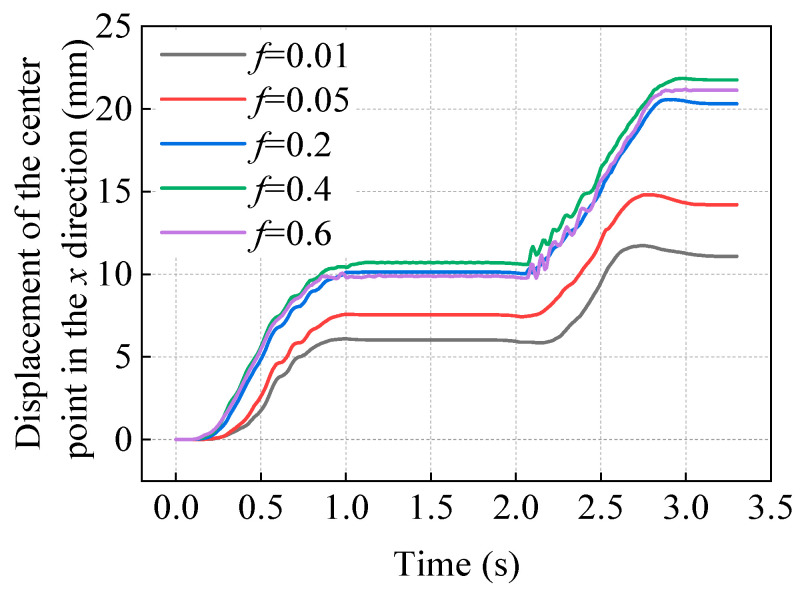
Time history of the displacement of the soft robot’s center point M in the *x* direction under different coefficients of friction.

To study the effect of the coefficient of friction on the motion of this soft robot, the measurement function of Comsol 6.1 software can be used to obtain the volume of the designed hydrogel soft robot as 670.54 mm^3^, the mass *m* as 7.91 × 10^−13^ kg/mm^3^, and the weight *T* as 7.75 × 10^−12^ N. The coefficient of friction between the soft robot and the contact surface is represented by *f*, and simulations are performed for one cycle of motion with *f* taken as 0.01, 0.05, 0.2, and 0.6 for comparison with the calculation results when *f* is 0.4 in the previous section (see Figure 20).

As can be seen from Figure 20, when the coefficient of friction *f* is less than 0.2, an increase in the coefficient of friction will significantly increase the distance that the soft robot can advance in one cycle. When the coefficient of friction *f* reaches 0.2, an increase in *f* will only slightly affect the distance that the soft robot can advance in one cycle. Among the several friction coefficients *f* tested, the soft robot advances the farthest in one cycle when *f* is 0.4, slightly more than when *f* is 0.6. This indicates that when the coefficient of friction is taken as 0.2, it can provide sufficient inertial force for the soft robot’s motion in the *z*-direction under the combined action of magnetic induction intensity and gravitational load, meaning that there is a critical friction coefficient for this soft robot’s motion model that ensures the frictional force can maintain the structural motion efficiency during its movement.

## 5. Conclusions

This paper introduces a correction factor representing the influence of magnetic domain density in the direction of magnetic flux density on the magnetic flux density into the magneto-mechanical coupled constitutive model, thereby improving the numerical calculation method for the dynamics of hard-magnetic particle hydrogel soft matter. The correctness of this method is verified by comparing it with theoretical solutions and experimental data. Furthermore, based on this method, a magnetically controlled hydrogel soft robot with a bipedal inchworm gait is designed, parameter optimization is conducted, and the influence of the coefficient of friction on the motion of the soft robot is analyzed. The research results show that under the fixed magnetic field load in this paper, the smaller the angle between the robot’s two feet, the higher its motion efficiency. However, an angle that is too small will cause self-contact between the two feet, hence a foot angle of 90° is ultimately chosen. At this time, the soft robot with a peak magnetic flux density load of 240 mT can advance 21.7 mm, equivalent to its foot length, in each cycle. The calculation results also indicate that there is a critical coefficient of friction of 0.2 for the soft robot presented in this paper. That is, when the coefficient of friction exceeds 0.2, its motion efficiency will not increase with the increase in the coefficient of friction, and when the coefficient of friction is below 0.2, its motion efficiency will increase with the increase of the coefficient of friction.

## Figures and Tables

**Table 1:** Material parameters of the magnetic hydrogel soft robot.

Parameter	Value
Shear modulus *G*	306 Kpa
Residual magnetic flux density **B**^r^	22.5 mT
Density	1.18 $g/{\mathrm{cm}}^{3}$
Coefficient of friction	0.4

## Data Availability

The data presented in this study are openly available in article.
